# Dynamic self-assembly and self-organized transport of magnetic micro-swimmers

**DOI:** 10.1038/s41598-017-15193-z

**Published:** 2017-11-07

**Authors:** Gašper Kokot, German V. Kolmakov, Igor S. Aranson, Alexey Snezhko

**Affiliations:** 1 Materials Science Division, Argonne National Laboratory, 9700 South Cass Avenue, Argonne, IL 60439 USA; 2 Physics Department, New York City College of Technology, the City University of New York, Brooklyn, NY 11201 USA; 3 Department of Biomedical Engineering, Pennsylvania State University, University Park, PA 16802 USA

## Abstract

We demonstrate experimentally and in computer simulations that magnetic microfloaters can self-organize into various functional structures while energized by an external alternating (ac) magnetic field. The structures exhibit self-propelled motion and an ability to carry a cargo along a pre-defined path. The morphology of the self-assembled swimmers is controlled by the frequency and amplitude of the magnetic field.

## Introduction

In the past decade, significant attention has been attracted to self-organization in active biomimetic materials such as interacting artificial cells^1,2^, synthetic spermatozoa^3^, autonomously driven microtubules^4^, self-locomoting gold-platinum (AuPt) nano-rods^5^, self-phoretic Janus particles^6^, and magnetic swimmers in aqueous solutions^7–14^. These and other self-propelled materials and synthetic systems transduce stored energy into their mechanical motion. Growing interests to the self-propelled matter is mainly driven by emerging engineering applications, such as targeted cargo transport by active swimmers^15,16^, swimmer-assisted parallel assembly of microstructures^17^, various bio-medical applications^18^. Unlike passive colloids, self-propelled particles have a propensity to form moving “active” molecules^19,20^. A few strategies were proposed including ultrasound activation^21^ and magnetic field-assisted assembly and guiding^22^. However, control of self-assembled structures and prediction of their emergent functionalities appears to be a challenge.

In this article, we demonstrate by dedicated experiments and computer simulations that magnetic microfloaters on the liquid surface exhibit a functionality reminiscent to that for living systems. Specifically, while being energized by an external oscillating (ac) magnetic field, the floaters self-organize into larger structures that are able to self-propel along the surface. The applied ac field is parallel to the fluid surface and, thus, only energizes the floaters whereas their collective emergent behavior is caused by the long-range magnetic and hydrodynamic interactions. In effect, the floaters can cooperate with each other, for example, by forming a stable swimming pair. The capillary interactions between the floaters and non-magnetic particles are harnessed for the pick-up function where the floater captures a cargo particle. By applying a weak horizontal magnetic field, one can navigate the resultant floater-cargo complex and transport the cargo along a pre-defined path.

## Results and Discussion

**Figure 1:**
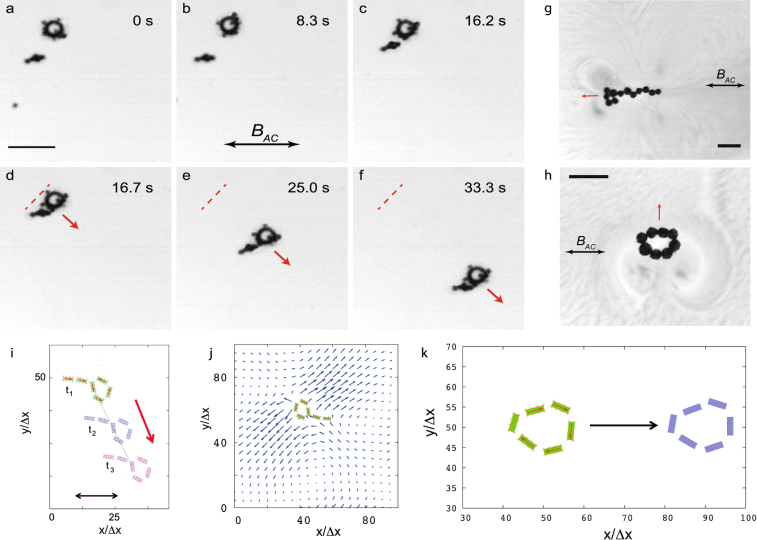
Self-assembly and autonomous motion of a magnetic swimmer on the surface of water. The energizing ac magnetic field is directed parallel the water surface. (**a**–**c**) Image sequence demonstrating the assembly process of two non-propelling structures. (**d**–**f**) Upon merging, the newly formed object exhibits directed motion. The red dashed line marks the initial position of the floater at the moment shown in (**d**). The ac magnetic field amplitude is ${B}_{AC}=1.01$ mT and the frequency is ${f}_{B}=15$ Hz. The field direction is shown by a horizontal double-headed arrow. The scale bar in (**a**) is 0.5 mm, the size of the magnetic particles, from which the swimmer was assembled, is 40 *μ*m. (**g**,**h**) Time-averaged hydrodynamic flows induced by a self-propelled magnetic “snake” and a ring. In both cases, the magnetic field axis was the same (double arrow line marked by ${B}_{AC}$), but the swimming directions were remarkably different, as indicated by red arrows. Scale bars are 0.5 mm, the particle size 120 *μ*m. (**i**) Graphical output from the numerical simulation of a self-assembled magnetic snake-like swimmer energized by an oscillating magnetic field for three subsequent moments of time. The numerical unit of length is ${\rm{\Delta }}x=20$ *μ*m. (**j**) The hydrodynamic flow around a swimmer simulated via our magnetic LSM-LBM. (k) Motion of a self-propelled magnetic ring energized by an oscillating magnetic field.

Our magnetic structures were self-assembled from a dispersion of nickel ferromagnetic microspheres that are freely floating on the surface of water. The particles were supported at the air-fluid interface by the surface tension. In our experiments, we observed that due to their magnetic interactions, the particles self-assembled into floating structures of various morphologies such as rings and rods^23–26^. Figure 1 shows representative results of our experiments with 15 magnetic particles. As it is seen in Fig. 1a, initially two clusters were formed: a ring and a rod-like object. At the later time, the rod approached the ring due to the magnetic attractive interactions between them and then, the two clusters join into a single elongated snake-like object (Fig. 1a–d). After the “snake” structure were formed, we turned on a homogeneous ac magnetic field $B={B}_{AC}\,\sin \,\mathrm{(2}\pi {f}_{B}t)$ applied parallel to the fluid surface (see Supplementary Information for more details). In response to the field, the structure swam along the fluid surface as it is seen in Fig. 1d–f. We also observed that other assemblies of floaters such as closed magnetic rings were able to self-propel if the ac magnetic field is applied. The direction of swimming depends on the morphology of the assembly and can be collinear (Fig. 1g) or perpendicular (Fig. 1h) to the direction of the magnetic field.

To understand the onset of magnetic self-propulsion we visualized the hydrodynamic flows by adding 2 *μ*m polymethyl methacrylate (pmma) microbeads to water. The result of the time-averaged flow visualization for a snake and for a closed asymmetric ring are shown in Fig. 1g,h. As it is seen in Fig. 1g, two pairs of counter-rotating vortices are created as the structure oscillates in the ac field. This vortex flow is the hallmark of force-free self-propelled objects (compare, e.g., to the flow pattern in Ref.^27^). The vortex structure around the ring in Fig. 1h is different from that for a snake in Fig. 1g since, unlike the snake, the ring self-propels due to breathing oscillations in an ac field. We emphasize that the applied ac magnetic field energizes the magnetic patterns, however it does not produce the net pushing force. The hydrodynamic force acting on the patterns vibrating in the field is the sole driving force that causes their translational motion along the fluid surface.

To further characterize the self-propelled motion of magnetic structures, we performed numerical simulations of the system based on the two-dimensional integrated lattice spring-lattice Boltzmann model (LSM-LBM), in which the magnetic interactions between the particles were taken into account (see Supplementary Information for details). To set-up the simulations, we positioned the magnetic particles on a grid with random orientation of the particles, run the simulations until the particles self-assemble into patterns, and then applied the ac magnetic field. Figure 1i shows the self-propelled motion of a self-assembled snake under the action of the ac field. Instants ${t}_{i}$ in Fig. 1i mark the initial ($i=1$) and subsequent ($i=\mathrm{2,3}$) moments of time; the red arrow shows the direction of swimming. Figure 1j visualizes the hydrodynamic flow around the snake swimmer averaged over a few ac field periods. An oscillating snake acts as a positive hydrodynamic dipole (a puller): it draws the fluid in the direction along its geometrical axis and ejects it is in the perpendicular direction. Due to the swimmer geometrical asymmetry, the net traction on the swimmer’s body due to the hydrodynamics flow is directed along the swimmer axis that results in the directed swimmer motion, as is seen in Fig. 1i. The numerical simulations also support our experiments of the propulsion of a self-assembled asymmetric ring, Fig. 1k.

**Figure 2:**
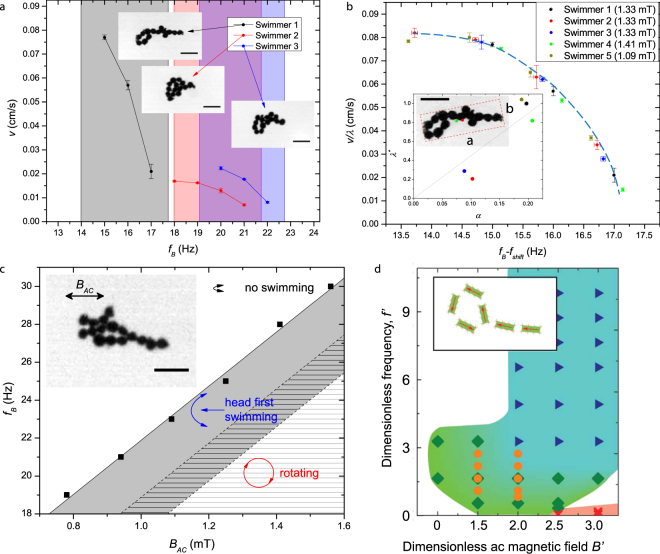
(**a**) Swimming speed $v$ vs magnetic field frequency ${f}_{B}$ for three self-assembled swimmers. The swimmer configurations are shown in the insets. The colored background marks the swimming region for a particular swimmer. The field amplitude is ${B}_{AC}=1.33$ mT. Scale bars in (**a**,**b**,**c**) are 0.5 mm, the particle size 120 *μ*m. (**b**) Swimming speed vs. ${f}_{B}$ for different swimmers collapsed onto a single universal curve. The dashed blue curve is a guide for the eye. Swimmer 1 was used as a reference and the other curves were shifted by ${f}_{shift}$ and scaled by the factor $\lambda$ to match the Swimmer 1 speed dependence. Inset: variation of the scaling factor ${\lambda }^{\ast }$ with the swimmer asymmetry $\alpha$, see the text. The line crossing the graph origin is a guide to the eye. The inset sketches the definition of the swimmer asymmetry. The green dot is the center of mass and the red dot is the geometric center of the bounding box (the dashed red line). The scale bar is 0.5 mm. (**c**) Experimental phase diagram of a typical swimmer composed of 120-*μ*m-in-diameter particles. The inset shows the swimmer, the scale bar is 0.5 mm. White region: the swimmer oscillates, but stays at rest. Gray region: the structure exhibit self-propelled motion. Shaded region: the structure develops rotational motion; self-propulsion is suppressed. The overlapping of the gray and shaded areas corresponds to the hysteresis behavior. (**d**) Computational phase diagram for a self-assembled snake-like magnetic swimmer. The snake’s shape is shown in the inset. Symbols show the parameters, for which the simulations have been done. The blue-green area corresponds to self-propulsion of a self-assembled magnetic snake. Green diamonds: the snake moves towards its head, blue triangles: the snake moves towards its tail. In the red-shaded region, the snake reconnects into a symmetric non-swimming ring. The orange circles show the parameters, for which an asymmetric ring shown Fig. 1k is motile.

We studied the dependence of the swimming velocity on the ac field frequency ${f}_{B}$, see Fig. 2a,b. The swimmers were self-assembled from the same set of 120-*μ*m-in-diameter spherical magnetic particles. Fig. 2a depicts the averaged swimmer speed $v$ vs the frequency ${f}_{B}$ for different swimmer’s shapes. It was found that for all swimmers, the velocity decreased with the rise of the frequency ${f}_{B}$. However, the self-propulsion frequency range was sensitive to the swimmer’s shape. The respective frequency ranges for three swimmers are marked by color shades in Fig. 2a.

We found that the dependence of the swimming speed $v$ on the frequency ${f}_{B}$ collapses into a single universal curve after ${f}_{B}$ is shifted by some reference frequency ${f}_{shift}$ and the speed $v$ is scaled by a dimensionless factor $\lambda$, as shown in the main plot in Fig. 2b. The universal curve in Fig. 2b describes the swimmers’ velocity in the entire range of frequencies, in which the swimmers are motile. As it is seen in the inset of Fig. 2b, the factor ${\lambda }^{\ast }\equiv \lambda ({B}_{AC}/{B}_{AC}^{\ast })$ scaled based on the ac field amplitude ${B}_{AC}$ is approximately proportional to the degree of geometrical asymmetry of a swimmer $\alpha$. The reference value ${B}_{AC}^{\ast }=1.33$ mT was taken equal to the field amplitude for swimmer 1 in Fig. 2a. The geometrical asymmetry $\alpha =\delta r/{A}^{\mathrm{1/2}}$ was defined as a ratio of the distance $\delta r=|{r}_{cm}-{r}_{geom}|$ between the center of mass ${r}_{cm}$ and the geometrical center of the structure ${r}_{geom}$, and a square root of the area $A=ab$ of the rectangular box enclosing the swimmer (see the inset in Fig. 2b). The longest side of the box is aligned with the swimming direction that makes this definition unique. The geometrical center ${r}_{geom}$ was determined as the box center. In accordance with our experiments, symmetrical objects, such as compact circular structures or linear chains, did not exhibit any self-propelled motion. This confirms our conclusion that the geometrical asymmetry is an important factor characterizing the directional propulsion of the structures. This is also consistent with Fig. 2b showing that more asymmetric configurations with higher $\alpha$ swim faster.

Figure 2c summarizes our experimental observations for a single snake-like swimmer. The phase diagram in Fig. 2c delineates different swimming regimes. The gray region corresponds to the parameters $({B}_{AC},{f}_{B})$ for which the swimmer exhibits self-propelled motion along the fluid surface. If the field frequency ${f}_{B}$ is high or the field amplitude ${B}_{AC}$ is low, the swimmer oscillates in response to the field but does not move along the surface (the white area). At high field amplitudes and low frequencies, the swimmer rotates and, again, does not exhibit any translational motion (the shaded area). The rotation of the swimmers in a low-frequency oscillating field is consistent with previous experiments of magnetic spinners^28,29^. At the boundary between the gray and shaded areas, the swimmers demonstrated a history-depended dynamics that is, the hysteresis. Specifically, if one increases the field frequency ${f}_{B}$ so that the point $({B}_{AC},{f}_{B})$ shifts from the shaded to hysteretic area on the phase diagram, the swimmer continues rotating. If the frequency was decreased from the gray to hysteretic area, the swimmer continues swimming.

We also constructed a numerical phase diagram for a snake swimmer, Fig. 2d. In the simulations, we characterized the ac field by its dimensionless amplitude $B^{\prime} ={B}_{AC}{L}_{0}^{3}/{\mu }_{0}M$ and dimensionless frequency $f^{\prime} ={L}_{0}^{2}{f}_{B}/\nu$ (Here, ${L}_{0}=90$ *μ*m is the magnetic particle size, ${\mu }_{0}$ is the magnetic permeability of free space, $M$ is the magnetic dipole moment of the particle, and $\nu$ is the viscosity of water.) The computational phase diagram in Fig. 2d for the magnetic snake is in qualitative agreement with our experiment, Fig. 2c (see Methods and Supplementary Information for the simulations details).

**Figure 3:**
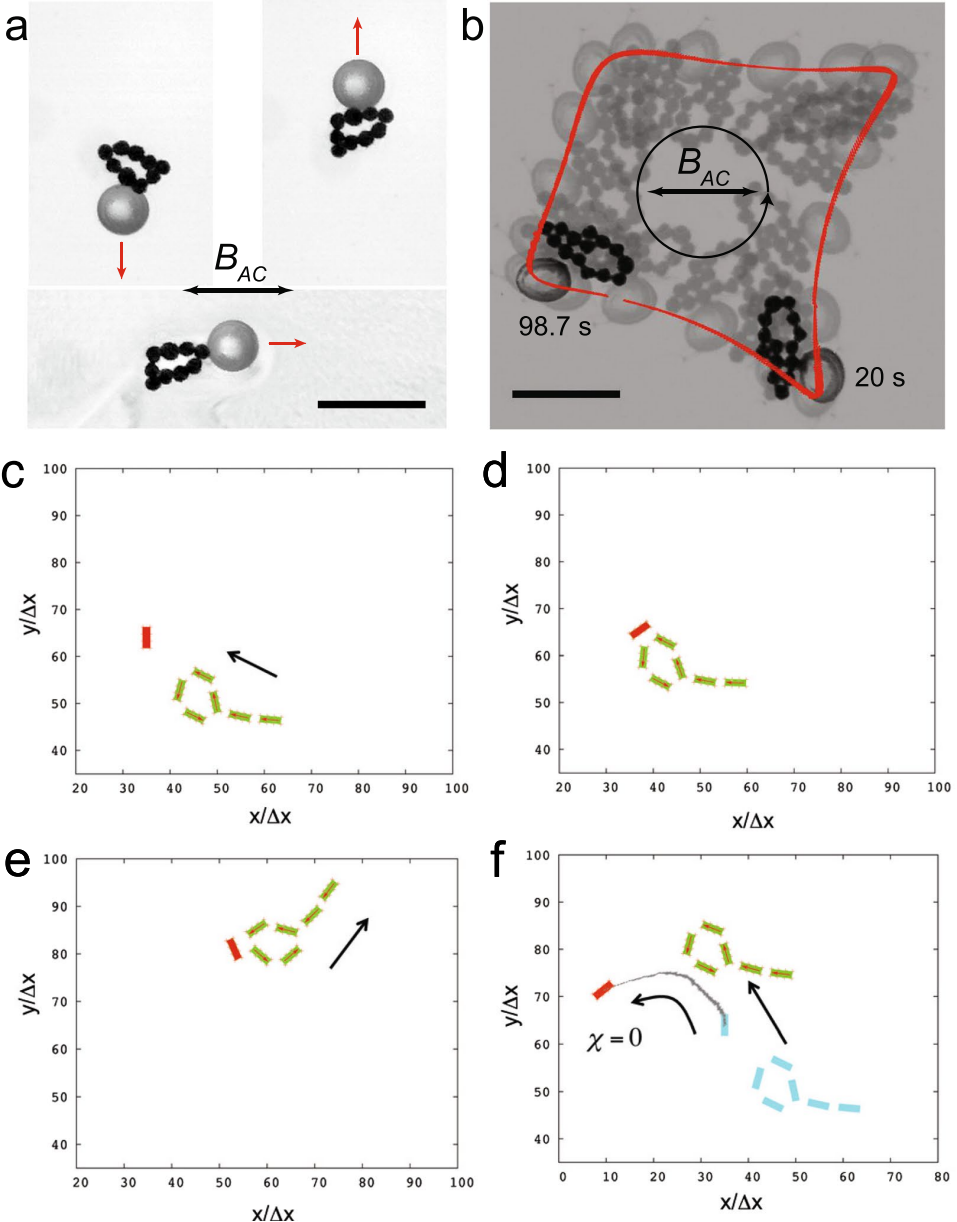
Directed transport of a cargo by a magnetic swimmer. (**a**) Self-propelled magnetic ring with an attached glass bead. The propulsion direction (red arrows) depends on the relative position of the cargo (the bead) and the swimmer (the ring). The ac magnetic field direction is shown as a double-headed arrow, ${B}_{AC}=1.17$ mT, ${f}_{B}=5$ Hz. (**b**) Steering of the cargo transport by changing the direction of the ac field. The image represents an overlay of several snapshots at different times. In this realization (${B}_{AC}=2.5$ mT, ${f}_{B}=10$ Hz), the field axis was steadily rotated at a frequency of 0.01 Hz. In effect, the swimmer carried the cargo on a closed loop (a red trace). The scale bars are 1 mm. The magnetic particle size is 120 *μ*m. (**c**–**e**) Graphical output from the simulations encompassing a self-propelled magnetic snake and a paramagnetic particle. The snake approaches the paramagnetic particle shown in red (**c**), picks it up (**d**) and then, transports it away from this location (**e**). After the paramagnetic particle attaches to the snake, the latter changes the swimming mode from head-first to tail-first swimming. In (**f**) the particle is magnetically neutral ($\chi =0$). In this case, the particle is eventually repelled from the snake by the hydrodynamic flow. The gray curve in (**f**) shows the trajectory of the particle. The initial positions of the snake and the particle are shown in blue. The numerical unit of length in the simulations is ${\rm{\Delta }}x=20$ *μ*m.

We showed that the magnetic swimmers can serve as carriers for the controlled transport on the fluid surface. In these experiments, we used a 0.5 mm glass bead as a “cargo” and a self-assembled ring as a “transporter”. The experimental results are shown in Fig. 3a,b. The glass bead was captured by the ring due to the capillary interactions^30,31^ and remained attached to the ring in the course of the expriment. As it is shown in Fig. 3a, the swimming direction of the ring-bead composite was determined by its spatial orientation, whereas the ac magnetic field only energized the swimmer without defining the direction of the motion. We were able to turn the swimmer by temporarily lowering the frequency ${f}_{B}$, thus forcing the swimmer to rotate, according to the phase diagram above. Then, the frequency was returned to its initial value and the swimmer continued its translational motion in the new direction. In the experiments with a ring-bead composite swimmer, the average swimming velocity was $v=0.013\pm 0.003$ cm/s that is close to the minimum velocity observed in the experiments with a free snake swimmer (cf. Fig. 2a).

The ring-bead composite swimmer can also be controllably stirred by the *variation in the direction* of the ac field. In these experiments, we slowly (at a rate of ${10}^{-2}$ Hz) rotated the ac field direction and kept the oscillation magnitude fixed. The results are shown in Fig. 3b. It is seen that the self-propelled ring with an attached bead moved along a closed path, made the full turn, and eventually returned to the position close to its initial position while the ac field rotated by 360°.

To gain insight into the swimmer-cargo dynamics, we modeled the system that encompassed a magnetic snake and a floating paramagnetic particle. The magnetic interactions between the ferromagnetic and paramagnetic particles were accounted in frames of the dipole-dipole approximation with the volumetric magnetic susceptibility of the paramagnetic particle $\chi =1$ ^3^. (We note that the capillary interactions between the particles were neglected in our two-dimensional simulations, but these interactions can be taken into account in a rigorous way in three-dimensional simulations^32,33^) The paramagnetic particle was positioned in front of a magnetic snake as shown in Fig. 3c and the snake was energized by the ac field. As it is indicated in Fig. 3d, the snake approached the paramagnetic cargo and, then, picked it up due to the mutual attractive interactions between the paramagnetic particle and the snake. At a later stage, the snake with a cargo continued swimming as a composite swimmer however, the direction of their motion was changed: the snake with an attached particle moved towards its tail (Fig. 3e). The change in the swimming mode is evidently caused by the increase of the total mass of the head after the load was picked up.

To ensure that attractive interaction with the paramagnetic cargo is important for the pick-up and transporting functions of the snake, we repeated the simulation, in which, however, the magnetic susceptibility $\chi$ of the cargo particle was set to zero. The simulations showed that in this case the particle was first, attracted by the snake due to the hydrodynamic flow directed towards the snake’s head (cf. Fig. 1j) and then, pushed away from the snake due to the hydrodynamics flow in the direction perpendicular to the snake motion (Fig. 3f).

**Figure 4:**
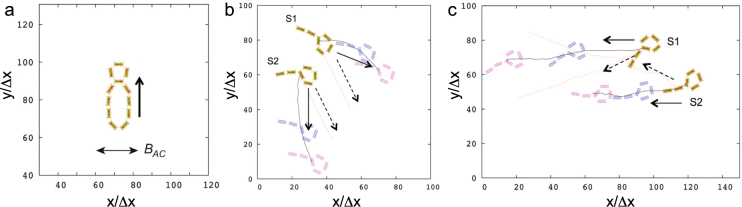
(**a**) A figure eight-shaped self-propelled swimmer. The directions of swimming and of the magnetic field ${B}_{AC}$ are shown by arrows. (**b**,**c**) Changes in the interaction of two snakes from repulsive to attractive with the change of the swimming mode. The system is energized at $f^{\prime} =1.64$ and $B^{\prime} =0.091$ in (**b**) and at $f^{\prime} =6.56$ and $B^{\prime} =0.121$ in (**c**). In panel (**b**) the snakes move towards their heads and repel each other. In panel (**c**), the snakes move towards their tails; the snake S2 follows the leading snake S1 due to attraction from a hydrodynamic wake formed behind S1. Dashed arrows show the directions of motion if only snake S1 or S2 is presented.

We also studied self-propulsion of more complex magnetic structures. Figure 1a shows a figure eight-shaped self-assembled pattern formed from two magnetic rings attached to each other. The shape of the object resembles a magnetic ring with an attached bead considered above (cf. Fig. 3a,b). As it is seen from Fig. 4a, in a close similarity with the latter, the eight-shaped pattern is able to swim in the direction perpendicular to the direction of the ac field.

Finally, we studied the interaction of two swimmers. For that purpose, we set up the simulations where two magnetic snakes, S1 and S2, were placed next to each other. The trajectories of the snakes and their subsequent positions for $f^{\prime} =1.64$ and $B^{\prime} =0.091$ are shown in Fig. 4b. It is seen that if two snakes are moving in the same direction towards their heads, their trajectories diverge. Thus, the snakes effectively repel each other. We also found that, if snakes were energized at the dimensionless frequency $f^{\prime} \gtrsim 2-3$, they moved towards their tails similar to that shown in Fig. 3e (c.f. the phase diagram in Fig. 2d). We have studied the interactions of two snakes in this regime as well. Two snakes, S1 and S2, were again placed next to each other and energized at $f^{\prime} =6.56$ and $B^{\prime} =0.121$, see Fig. 4c. In contrast to the simulations at low frequencies (Fig. 4b), snake S2 follows snake S1, as demonstrated by their nearly parallel trajectories (black curves and solid arrows). It is also seen that the trajectories of both snakes differ from those obtained in the case where only snake S1 or S2 is present (pink curves and dashed arrows). The reason for the difference in the snakes' behavior at low and high frequencies can be understood as follows. Since both snakes are aligned in the above simulations and, hence, their total magnetic moment were approximately collinear, the net magnetic interactions of the snakes were repulsive. (We note that the magnetic moments of the particles in the snakes' tails were directed from the head to the tail end for both snakes, and the net moment of closed rings, which forms the heads, is negligible. Thus, the snakes can be viewed to a first approximation as two linear magnets.) While being energized by the low-frequency driving field at low ac field amplitude, for which the snakes move toward their heads, the snakes speeds are relatively small and thus, the effect of hydrodynamic interactions between the snakes is weak. In this case, the magnetic repulsion dominates, and their trajectory diverge, as is seen in Fig. 4b. In the opposite case of high-frequency high-field driving (Fig. 4c), the snakes' speeds are increased in a few times, and the hydrodynamic interactions between the snakes dominates. In effect, snake S2 in Fig. 4c was attracted by the fluid motion in the hydrodynamic wake formed behind the leading snake S1 and then, it followed the latter. We note that the correlations and bound pair formation due to the hydrodynamic interactions for a swimming pair was analyzed in Ref.^34^.

## Conclusions

In conclusion, by combining experiment and simulations, we have studied self-organization in the system of magnetic particles into complex functional assemblies. We investigated how the structures exhibit self-propelled motion, and studied their collective dynamics. We have also shown that the self-propelled structures can be effectively used as versatile transport agents that can capture and, then, carry their cargo in a desired direction defined by the applied ac field.

In our studies we were inspired by similarities between the behavior of self-propelled synthetic systems and living microorganisms, including the examples mentioned in the introduction. One specific example is given by motile magnetotactic bacteria that can navigate in the Earth’s geomagnetic field by using magnetosomes, nanometer-sized crystals of magnetic iron which play a role of the nano-size compasses.^35,36^. Motile magnetitactic bacteria have attracted attention due to a number of potential applications in environmental science^37,38^, and non-destructive soft materials analyses^39^. In particular, it was demonstrated that magnetosomes in aqueous solutions can collectively work as immobilizers of bioactive substances, which can then be transported by magnetic fields^36^. We were also inspired by potential application of biomimetic microsystems in medical applications such as targeted drug delivery for antitumor treatments^40^, as a contrast agent for magnetic resonance imaging (MRI)^41^, and as micro-robots for minimal-invasive surgery^42^. The goal of our studies is to find pathways toward the implementation of these functionalities in biomimetic self-assembled motile magnetic microswimmers.

The next logical step would be to study the controlled assembly of microswimmers optimized for specific functions including those listed above. One of the possible ways is to utilize shaped microparticles such as rods with tunable aspect ratio^43^ or lock-and-key colloid dimers and polymers with complimentary geometrical properties^44,45^. Potentially, variation in geometry and magnetic properties of the building blocks will enable one to engineer self-assembled swimmers with desired functionalities, controlled motility and collective (magnetic and hydrodynamic) interactions.

## Methods

### Experimental

To assemble the floaters, two sets of nickel (Ni) ferromagnetic microparticles with average diameter of 40 *μ*m and 120 *μ*m were used. The particles were suspended at the water-air interface inside a glass beaker 5 cm in diameter mounted at the microscope stage (Leica MZ9.5). The experiments reported were carried out with about 15 particles. In most experiments, the particle ensemble was energized by a uni-axial oscillating magnetic field $B={B}_{AC}\,\sin \,\mathrm{(2}\pi {f}_{B}t)$ applied in-plane with the water-air interface. The spatially uniform magnetic field in the beaker was generated by a pair of custom made coils (Helmholtz configuration). In the experiments where we demonstrated the possibility to navigate the propulsion with by changing the magnetic field direction, Fig. 3b, a bi-axial magnetic field setup was used. The bi-axial field was generated by Evico Magnetics coils. The dynamics of the swimmers were monitored by a fast complementary metal-oxide-semiconductor (CMOS) camera iNS1 (Integrated Design Tools Inc). The numerical package ImageJ and custom codes have been used for the image processing and to track the swimmer position. The flows induced by swimmers were visualized with the help of nonmagnetic tracers 2 *μ*m pmma microparticle (Bangs Laboratories Inc.) suspended in the fluid near the surface. The cargo particle carried by a magnetic ring in Fig. 3a,b was a non-magnetic glass particle $\sim 0.5$ mm in diameter made by Ceroglass Technologies Inc.

Ferromagnetic nature of the nickel particles leads to the aggregation into a branched cluster in the absence of an external magnetic field. An external magnetic field can be used to modify the morphology of the static cluster. To rearrange the structure into a swimmer the particle ensemble was driven into a spinner state^28^ then the oscillating field was turned off and the self-assembled structure was assessed for swimming capabilities.

### Simulations

The particles dynamics was captured by the lattice spring model (LSM), which consists of point masses connected by a network of Hookean springs^46^. The square LSM lattice described isotropic elastic solid with Young’s modulus $E=5{k}_{l}/2{\rm{\Delta }}{x}_{LSM}$ where ${\rm{\Delta }}{x}_{LSM}$ is the LSM lattice constant and ${k}_{l}$ is the rigidity of diagonal springs^47,48^.

The fluid dynamics and the fluid-structure interactions are described by the lattice Boltzmann model (LBM) coupled with the LSM.^47,49^. An integrated LSM-LBM model was earlier utilized in the studies of microcapsules immersed in a fluid^47,50^, self-propelled motion of communicating artificial cells^2^, biomimetic cilia^51^, and a flexible filament mimicking artificial spermatozoa^52^.

We consider the particles moving in a shallow fluid layer and thus, we utilize the two-dimensional (2D) LSM-LBM model. To account for the finite fluid depth in the vertical direction we modify the 2D LBM model and incorporated the shallow-water approximation. The latter describes additional relaxation of the linear momentum of the fluid in the horizontal plane due to the viscous drag at the rigid bottom. The relaxation rate ${\gamma }_{h}=(3/2)\eta /{h}^{2}\rho$ depends on the fluid layer depth $h$. (Here, $\eta$ and $\rho$ are the respective viscosity and density of the fluid.)

The magnetic interactions between the particles were described in the magnetic dipole approximation. The details of the simulation model and the match between the experimental and simulations parameters are described in Supplementary Information.

### Data Availability

The generated data are available from the authors upon request.

## Electronic supplementary material

Supplementary Information
