# Supplementary material for: Dynamic self-assembly and self-organized transport of magnetic micro-swimmers
Source: Sci Rep. 2017 Nov 7;7:14726. doi: 10.1038/s41598-017-15193-z (PMC5677019; doi:10.1038/s41598-017-15193-z)
Supplement: Supplementary file 1 — Supplementary Information [file 41598_2017_15193_MOESM1_ESM.pdf]

Dynamic self-assembly and self-organized transport of synthetic micro-swimmers

Supplementary Information

Gasper Kokot,[a] German V. Kolmakov,[b] Igor S. Aranson,[ac] and Alexey Snezhko[a]

[a]*Materials Science Division, Argonne National Laboratory, Argonne, IL 60439, USA*
[b]*NYC College of Technology, the City University of New York, Brooklyn, NY 11201, USA*
[c]*Department of Biomedical Engineering, Pennsylvania State University, University Park, PA 16802, USA*

I. NUMERICAL MODEL

In the simulations elongated magnetic particles of a few tens micrometers size, immersed in a fluid, were our fundamental structural units (Fig. 1). These particles can be thought as a model of tightly bound pairs or triplets of magnetic particles in our experiments. Elongated particles can also be a model for ellipsoidal particles in synthetic colloids,[1,2] composite dimers,[3,4] extensile microtubule bundles,[5] or synthetic rods.[6]

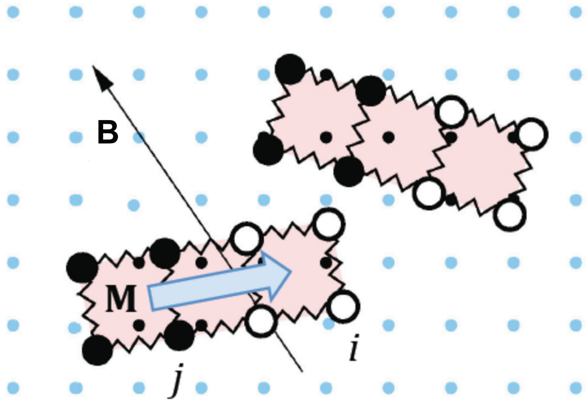

FIG. 1. Schematics of our system. The lattice spring nodes are connected by Hookean springs. The diagonal springs that connect each nodes with all its next-nearest neighbors are not shown). The swimmers are in the magnetic field **B**, which is a sum of the external field and the field created by other swimmers. The ith LSM nodes (unfilled circles) and jth nodes (filled circles) contribute to the north and south magnetic poles of the swimmer's magnetic moment **M**, respectively. The fluid LBM nodes (blue points) contain fluid particles. The solid LBM nodes inside the swimmers (black points) do not contain fluid.

The particles dynamics was captured by the lattice spring model (LSM), which consists of point masses connected by a network of Hookean springs (see schematic in Fig. 1).[7] The fluid dynamics and its interactions with the structures is described by the lattice Boltzmann model (LBM) coupled with the LSM.[8,9] An integrated LSM-LBM model was earlier utilized in the studies of microcapsules immersed in a fluid,[9,10] self-propelled motion of communicating artificial cells,[11] biomimetic cilia,[12] and a flexible filament mimicking artificial spermatozoa.[13]

In our simulations we consider the particles moving in a shallow fluid layer and thus, we utilize the two-dimensional (2D) LSM-LBM model. The finite fluid depth in the vertical direction was incorporated by using the shallow-water approximation. The latter describes additional relaxation

of the linear momentum of the fluid in the horizontal plane due to the viscous drag at the rigid bottom of the layer walls. The respective relaxation rate $\gamma_h = \frac{3}{2}\frac{\eta}{h^2\rho}$ depends on the fluid layer depth h. (η and ρ are respective viscosity and density of the fluid.)

In most simulations, the size of the computation box was set equal to $L_x \times L_y = 100 \times 100(\Delta x)^2$ (with Δx being the LBM spacing). In the simulations with two patterns, we increased one of the box dimensions to $150\Delta x$ as detailed below. The periodic conditions for the fluid flows were applied at the boundaries of the computational box. The fluid-structure interactions were captured via the linked bounce-back rule[9,14] at the LBM-LSM interface. To eliminate the short-wave instability in the LBM fluid simulations with immersed moving particles, the fluid velocities and forces acting on the LSM nodes have been averaged over two subsequent iterations at each numerical step.[14]

The square LSM lattice described isotropic elastic solid with Young's modulus $E = 5k_l/2\Delta x_{LSM}$ where Δx_{LSM} is the LSM lattice constant and k_l is the rigidity of diagonal springs.[9,15] In the simulations, we considered stiff particles, for which $\xi = \eta v/EL_0 \ll 1$ (where η is the dynamic viscosity of the fluid, and v and L_0 are the respective particle velocity and length), and thus, motion in the fluid did not result in mechanical deformations. We checked that in our simulations $\xi < 10^{-5}$.

The magnetic dipole moment of ferromagnetic particles was calculated from the positions of the LSM nodes as

$$\mathbf{M} = M_0 \left(\sum_i \mathbf{r}_i - \sum_j \mathbf{r}_j \right) / \Delta x_{LSM} \tag{1}$$

where M_0 is proportional to the total dipole moment of the particle, and the sums were taken for the radius-vectors $\mathbf{r}$ of the LSM nodes contributing to the north (ith) and south (jth) poles of the magnetic dipole (Fig. 1). The magnetic interaction between the ferromagnetic particles particles was captured in the dipole approximation.[16] This approximation gives the accuracy $\sim 90\%$ for the distance between the particles $\sim$ twice their size and better than 98% at distances larger than seven particle sizes.[17] The magnetic torque on the particles was calculated as

$$\mathbf{K} = [\mathbf{M} \times \mathbf{B}]. \tag{2}$$

where the net field $\mathbf{B}$ was the sum of the external energizing ac field and the field created by magnetic particles.

For paramagnetic particles in the magnetic field $\mathbf{B}$, the magnetic moment was calculated as

$$\mathbf{M} = \chi \left[(\mathbf{B} \cdot \hat{\mathbf{r}}_a)\hat{\mathbf{r}}_a/n^a + (\mathbf{B} \cdot \hat{\mathbf{r}}_b)\hat{\mathbf{r}}_b/n^b \right], \tag{3}$$

where $\hat{\mathbf{r}}_{a(b)}$ are the unit vectors directed along (perpendicular to) the particle. The respective demagnetizing factors $n^{a(b)}$ were found by approximating the particles as prolate magnetic ellipsoids with a two-dimensional volume $L_0\Delta L$ and the ratio of semi-principal axes length $\Delta L/L_0$.[18] (Here, $\Delta L \equiv \Delta x_{LSM}$ is the width of the particles.) The dipole magnetic moments of paramagnetic particles were relaxed at each numerical time step with the relative accuracy better than 10^{-5} by means of multiple iterations at fixed positions of the LSM nodes. In the simulations, we set the magnetic susceptibility of paramagnetic particles equal to $\chi = 1$.[19]

II. SIMULATION PARAMETERS

In the simulations, the spatial discretization of the LBM model was $\Delta x = 20$ μm and the numerical units of time was $\Delta t = 6.67 \times 10^{-5}$ s. The results of the simulations are presented for

the fluid layer depth $h = 1$ mm. However, we found that variations of the fluid depth h from 1 cm to 0.1 mm did not lead to any qualitative changes in the simulation results.

We characterized the driving frequency f_B of the energizing magnetic field by the dimensionless frequency $f' = L_0^2 f_B/\nu$. The kinematic viscosity of the aqueous solution was taken equal to $\nu \equiv \eta/\rho = 0.010$ cm^2/s where $\rho = 1$ g/cm^3 is the density of the aqueous solution. In the simulations, we vary the dimensional frequency in the range $f' = 0.01 - 10$. This range corresponds to the conditions of our experiment for the frequency range $f_B = 5 - 300$ Hz, for which $f' \sim 0.04 - 2.4$. The particle size was taken equal to $L_0 = 90$ μm. It is consistent with our experiments with magnetic particles described in the main text. Our parameters are also relevant for experiments with a motile magnetic filament of length ~ 30 μm attached to a human red blood cell[19] and driven at the frequency of ~ 10 Hz; in the latter case, one has $f' \sim 0.01$. It is also worth noting that the respective dimensionless frequency for motile human sperm (the length is ~ 55 μm, the beat frequency is $10 - 20$ Hz, ref. 20) is $f' \sim 0.03 - 0.06$ that again lies in the same range.

The external energizing magnetic field strength, B_{AC}, was characterized in the simulations via the dimensionless magnetic number $\mathrm{B} = B_{AC} L_0^3/\mu_0 M$, where M is the magnetic dipole moment of the particle (with $\mu_0 = 4\pi \times 10^{-7}$ NA^{-2} being the magnetic permeability of free space). The dimensionless magnetic field B can be viewed as a ratio of the magnetic torque on a particle due to the interaction with the external field, $\tau_{\text{ext}} \sim MH_0$, and of the torque produced by the dipole interaction with a neighboring particle separated by a distance L_0, $\tau \sim \mu_0 M^2/L_0^3$. In the simulations, we set $M = 20$ μemu (ref. 21) and varied the dimensionless field strength in the range $\mathrm{B} = 0.06 - 0.20$. This range approximates the interval of the magnetic fields $B_{AC} = 10 - 60$ Oe in our experiments and in Ref. 21, for which one has $\mathrm{B} \approx 0.03 - 0.18$.

To further characterize the conditions, at which the self-assembled magnetic structures are motile, we also estimated the so-called sperm number $\mathrm{Sp} = L_0 \left(\frac{2\pi f \gamma^{\perp}}{G}\right)^{1/4}$ that characterizes the relative importance of viscosity compared to bending forces.[19] Here, G stands for the bending modulus of the structure, and $\gamma^{\perp}$ is the coefficient of the viscous drag in the direction perpendicular to the structure.[22] We determined the bending modulus G of the filament numerically via the slope of the stress-strain curve for four-point bending deformation[23] of a chain of two magnetically coupled, aligned particles. The perpendicular viscous drag coefficient was taken equal to $\gamma^{\perp} = 4\pi\eta$.[22,24,25] In our simulations for motile snake-like structures (the colored region in the phase diagram in Fig. 2d in the main text), the characteristic value for the sperm number is $\mathrm{Sp} \sim 5 - 6$.

For a single-armed swimmer, the optimal conditions for the motility is $\mathrm{Sp} \sim 1$ (ref. 22) whereas for $\mathrm{Sp} \to 0$ swimmers are not motile because the time-reversal symmetry is restored in this limit.[26] An increased value $\mathrm{Sp} = 5 - 6$ for motile structures in an external field, compared to $\mathrm{Sp} = 1$, is in agreement with the observations[19] made for a magnetic field-driven DNA-linked filament. The sperm number in our simulations is also close to $\mathrm{Sp} \approx 7$ for motile human spermatozoa.[19]

III. FORMATION OF MAGNETIC STRUCTURES

In our simulations, we, first, considered the formation of magnetic structures from loose particles. For this purpose, the particles ($L_0 = 90$ μm in length, $\Delta L = 30$ μm in width) were arranged in a square lattice with random orientations in the (x, y) plane (Fig. 2a). In the simulations, we vary the dimensionless particle density in the simulation box, $n = N(\Delta x)^2/L_x L_y$, in the limits $n = (1.6 - 3.6) \times 10^{-3}$ (where N is the number of particles and Δx is the spatial discretization of the numerical LBM grid) that corresponds to the volume fraction of particles $\phi \equiv V_0 N/L_x L_y = 0.011 - 0.024$ (where $V_0 = L_0 \Delta L$ is the 2D volume of a single particle). Thus, our system is in the regime where loose magnetic structures can form.[27–30]

An example of the temporal evolution of the system is shown in Figs. 2a,b. We found that, by the time $t \sim 10^5 \Delta t$, a few ultimate patterns are formed that can be related to the following four classes: (A) straight or curved strings; (B) closed rings; (C) snakes, or "spermatozoa" that encompass a head in a form of a ring with a "tail" attached to it; and (D) complex structures with higher connectivity that are not related to classes A-C. A histogram in Fig. 2c shows the relative, averaged fraction number of structures A-D formed in the simulations. It is seen that $\sim 29\%$ of particles are assembled in snake-like patterns C, and $\sim 15\%$ of particles form closed rings. (We note that Fig. 2c only shows examples of the patterns whereas the actual number of particles in each pattern can vary; for example, we found that, at given particle densities, rings can encompass four to seven particles.) We also found that the presence of a uniform, time-independent magnetic field in the (x, y) plane supresses the pattern formation; instead, the particles tend to orient along the field and only form linear structures A of different length.

IV. SELF-PROPULSION OF LOOSE PATTERNS ENERGIZED BY AN A.C. MAGNETIC FIELD

We tested the motility of various patterns formed in the above simulations in the presence of an a.c. magnetic field. Specifically, we isolated the patterns, and applied the field $\mathbf{B}(t) = \mathbf{B}_{AC} \sin(2\pi f_B t)$ in the (x, y) plane.

Our simulations showed that open strings (A) oscillate in response of the *a.c.* magnetic field however, they did not demonstrate any motility. In contrast, snakes (B) energized by the *a.c.* show self-propelled motion with the average speed depending on the field magnitude B_{AC} and frequency f; the examples are shown in Figs. 3a,b. It is seen that there are two modes of swimming: (i) the snake moves in the direction of its head (Fig. 3a), and (ii) the snake moves in the direction of its tail (Fig. 3b).

To understand the reason for switching between the two modes, we analyzed the snake dynamics in greater details. It was found that, depending on the a.c. field magnitude and frequency, four general scenario exist. First, at very low magnetic fields or frequency, the snake periodically rotates as a whole in response to the excitation field oscillations however, its mean displacement is negligible (the dimensionless speed, determined via the displacement of the snake's center of

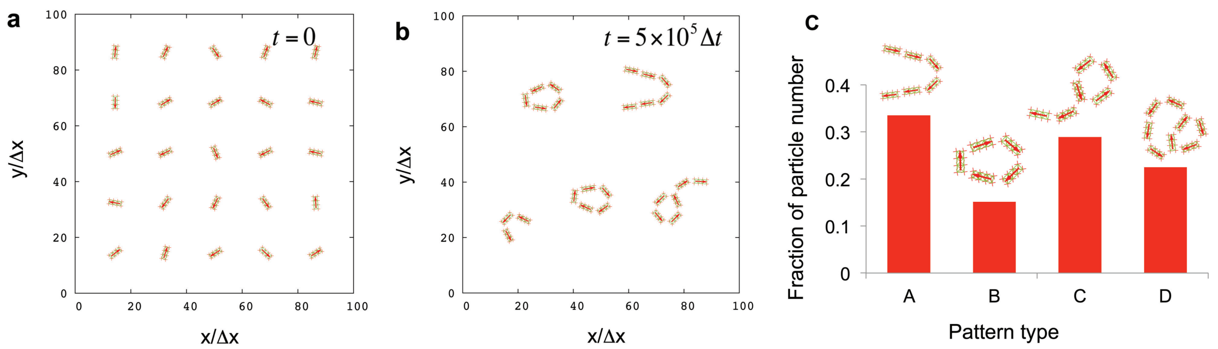

FIG. 2. Formation of magnetic patterns from loose particles: figure (**a**) shows the initial conditions at time $t = 0$ and figure (**b**) shows the particle configuration formed by the moment $t = 5 \times 10^5 \Delta t$. The simulation box is $100 \times 100(\Delta x)^2$. Red arrows on the particles show the direction of their magnetic dipole moments. A histogram in (**c**) shows the fraction of the number of particles that are self-assembled in patterns A-D by the moment $t = 5 \times 10^5 \Delta t$. The data in the histogram is averaged over seven independent realizations. Examples of the patterns formed are shown above the bars.

mass, is $v/L_0f < 8 \times 10^{-5}$ that corresponds to $v < 1$ μm/s for 90-μm particles). When the field magnitude or frequency rise, the snake's tail begins producing wide, large amplitude oscillations with relatively large deformation and with the beat frequency of $\sim f/3 - f/4$ (Fig. 3c). If the field magnitude or frequency rise even further, the mode of oscillations changes again: the amplitude of the over-all tail displacement is decreased, compared to the previous large-amplitude mode; the particles mostly rotate around their centers of mass whereas the displacement of the particle center of mass displacement is less than the particle size (Fig. 3d). For large-field low-frequency drivings, the snake becomes unstable and is reconnected to a symmetric ring; the latter does not show any motility if being energized by the field.

Our results are summarizes on the phase diagram in Fig. 2d in the main text. At the crossover between the swimming regimes (i) (green diamonds on the numerical phase diagram in the main

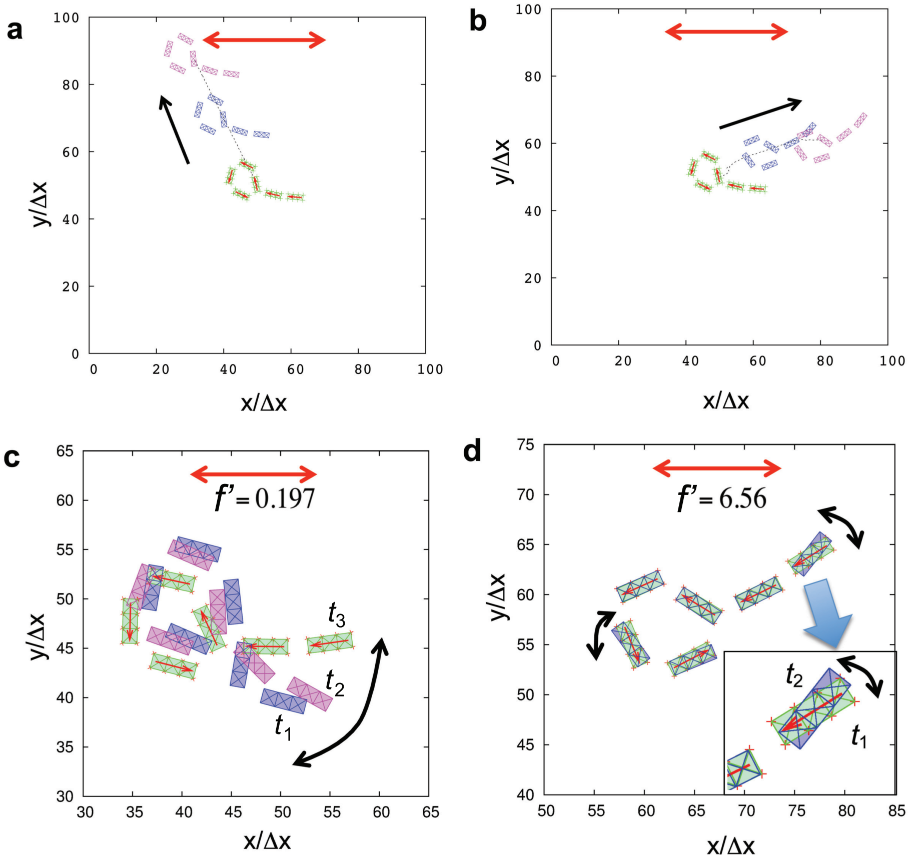

FIG. 3. (a,b) Motion of a self-assembled magnetic snake energized by an a.c. field. (a): The snake moves towards its head for low field magnitude and frequency, B = 0.091 and $f' = 1.64$. (b) In higher field magnitude and frequency, B = 0.121 and $f' = 6.56$, the character of its motion is changed and the snake moves towards its tail. (c,d): Changes in the snake motion with rising the ac field frequency from $f' = 0.197$ (c) to $f = 6.56$ (d). It is seen that at low frequencies (c), the snake's tail and head make large amplitude oscillations with the displacement of the order of the particle size. The positions of the particles are shown for $t_1 = 5.920 \times 10^4 \Delta t$ (blue shade), $t_2 = 6.056 \times 10^4 \Delta t$ (pink), and $t_3 = 6.320 \times 10^4 \Delta t$ (green). In contrast, for high frequency (d) individual particles rotate around their centers of mass, whereas the over-all tail and head displacements are small. The inset in (d) shows the particle oscillations in the tail of the snake for $t_1 = 90046.7\Delta t$ (green shade) and $t_2 = 90089.9\Delta t$ (blue). The dimensionless driving amplitude is B = 0.091 for figure (c) and $B = 0.121$ for (d). The horizontal red arrow shows the direction of the a.c. driving field in (a)-(d).

text) and (ii) (blue triangles on the phase diagram), the snake shows less-directed motion; for example, it can start swimming, first, toward its head and then, turn perpendicularly to its initial direction of motion. We found that the dimensionless velocity of a motile snake is a slowly decreasing function of the field frequency. For example, for the driving field magnitude B = 0.12, the snake's dimensionless velocity falls from $v/L_0 f = 8 \times 10^{-3}$ to 5×10^{-3} when the dimensionless frequency rises by approximately an order of magnitude, from $f' = 0.16$ to 1.1. The physical velocity of the snake, however, increases at the same time from ≈ 15 μm/s to 65 μm/s. We note that the obtained velocities are of the same order as for human spermatozoa is an aqueous solution ($25 - 45$ μm/s, ref. 20). The snake velocity is also close to the velocity of paramagnetic filament (~ 25 μm/s) in mT-strong oscillating field in ref. 19.

We also demonstrate that one can effectively control the swimming direction of the snake by choosing an appropriate driving mode. For this purpose, we setup the simulations where the snake was, first, energized by a low-field, low-frequency driving (B = 0.091, $f' = 2.19$), at which it exhibits the motion of type (i). Then, at the moment $t_{sw} = 4.5 \times 10^5 \Delta t$, we switch the field magnitude and frequency in a step-like manner to B = 0.12, $f' = 9.84$ that correspond to the motion of type (ii). Fig. 4 shows the graphical output from this simulation. It is seen that at the moment t_{sw}, the snake changes its swimming direction to opposite and, first, returns back to its approximate initial position and then, continues to swim in this new direction.

We found that symmetrically assembled magnetic rings were not motile. However, asymmetric rings self-propelled in the fluid while being energized by an ac field, as shown in Fig. 1k in the main text.

V. INTERACTIONS OF MOTILE MAGNETIC STRUCTURES

To understand the collective dynamics of self-assembled magnetic structures, we studied the interactions of motile patterns energized by an external field. We focus primarily on two snakes to gain insight into the interplay between the hydrodynamic and magnetic interactions of the patterns. The results for two snakes moving in the same direction are reported in the main text and shown in Fig. 5b,c there.

We also studied the cases where two snakes were heading toward each other. We found that, in

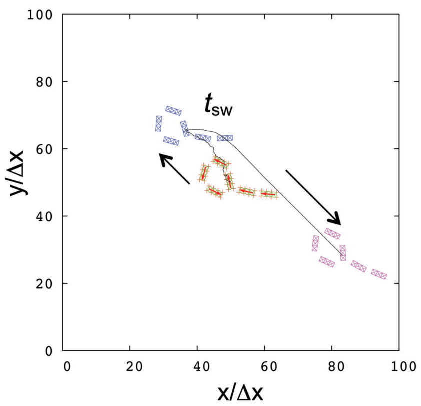

FIG. 4. Control of the swimming direction of the magnetic snake by changing the energizing field. Initially, the snake was energized by a low-field low-frequency driving (B = 0.091, $f' = 2.19$), at which it moved towards it head. Then, at the moment $t_{sw} = 4.5 \times 10^5 \Delta t$, the field magnitude and frequency was switched in a step-like manner to B = 0.12, $f' = 9.84$ that correspond to the motion towards the snake's tail.

a general situation, the snakes moving toward their heads "tend" to avoid each other; thus, they reproduced their repulsive behavior demonstrated in the simulations in Fig. 5b in the main text. In most cases, the snakes moving towards their tails also showed similar repulsive behavior if launched from arbitrary initial positions far from each other. However, if two snakes were directed toward each other during the onset of the simulations, they could collide and merge into a larger, motile pattern. Figs. 5a-d show an example of such interactions. The snakes where initially arranged in a tail-to-tail configuration, Fig. 5a, and energized at $f' = 6.56$ and B $= 0.121$. It is seen that the two snakes first, approached each other and produced a coupled pair (Fig. 5b) and then, they reconnected to a larger snake with six-particle head and a tail (Fig. 5c). The large snake continues its motion toward its tail as is depicted in Figs. 5c,d. It is also seen that, in the traveling large snake, two particles were eventually pushed out of the ring forming the head and eventually the head acquired the four-particle shape.

To compare the motility of different structures, we set a "race" where a ring with a paramagnetic inset competes with a snake with an attached cargo. For that, we arranged the two patterns next to each other, as shown in Fig. 6, and applied the energizing a.c. field. The parameters for the energizing field was chosen so that both the structures swim in the same direction, from right to left in Fig. 6. (Specifically, we set B $= 0.122$ and $f' = 3.28$ in the simulations shown in the figure.) It is seen that at $t = 2.26 \times 10^5 \Delta t$ after the field was turned on, the displacement of the ring is $\approx 2\times$ larger than that for the snake. The dimensionless velocity of the ring obtained in the simulation was $v' \equiv v/f_B L_0 \approx 1.10 \times 10^{-2}$ whereas for the snake with a payload it was $v' \approx 5.48 \times 10^{-3}$. (The actual velocities of the patterns were $v = 88.7$ μm/s and 44.3 μm/s, respectively). Thus, the

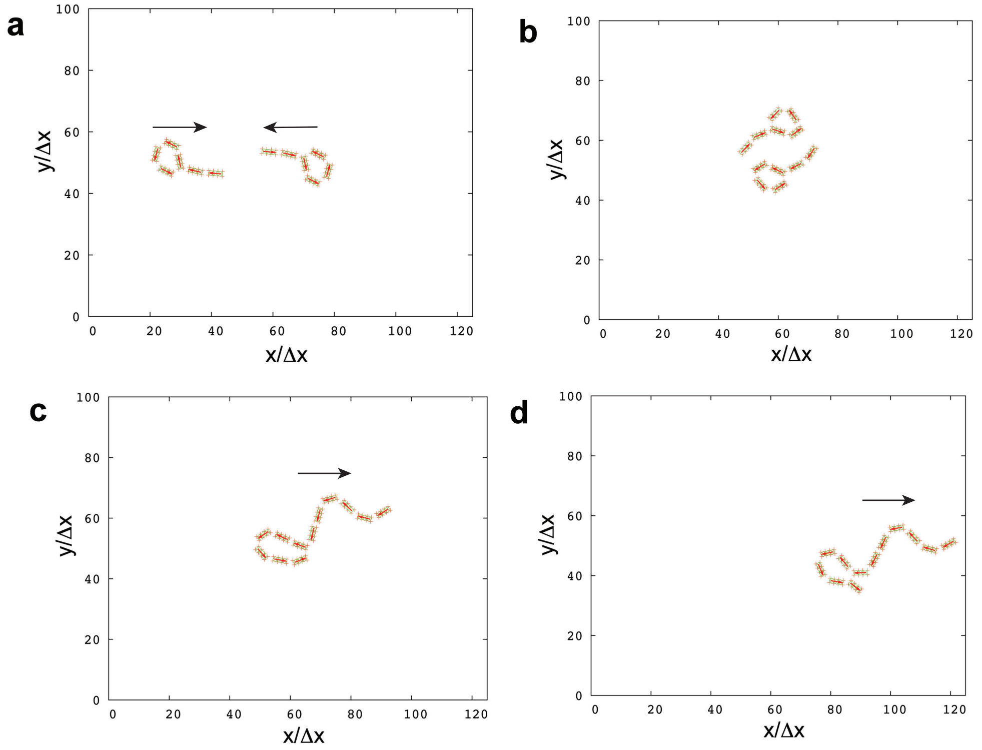

FIG. 5. Collision of two swimming magnetic snakes. The collision results in their merging into a larger motile snake. The frames (a-d) show the system in four subsequent moments of time. The system is energized at $f' = 6.56$ and B $= 0.121$. The energizing ac field is directed along the x axis. The arrows in (a), (c) and (d) mark the direction of swimming.

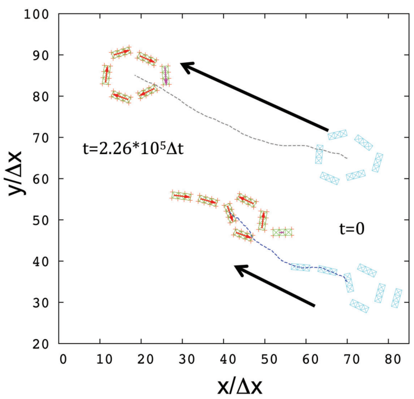

FIG. 6. The race between a self-assembled asymmetric ring with a paramagnetic inset and a snake with an attached cargo. It is seen that, by the moment $t = 2.26 \times 10^5 \Delta t$, the displacement of the ring is about two times larger than that for the snake. The energizing a.c. field is directed along the horizontal x axis.

efficiency, understood as fast delivery of a cargo by a carrier to a desired location, is higher for a ring-like structure. However, a snake provides better flexibility of the functions, including pick up and delivery functionality and the possibility to control the direction of the carrier motion by choosing the appropriate parameters of the energizing field.

---

[1] Han, Y. *et al.* Brownian motion of an ellipsoid. *Science* **314**, 626–630 (2006).

[2] Kuijk, A., van Blaaderen, A. & Imhof, A. Synthesis of monodisperse, rodlike silica colloids with tunable aspect ratio. *J. Am. Chem. Soc.* **133**, 2346–2349 (2011).

[3] Sacanna, S., Irvine, W. T. M., Chaikin, P. M. & Pine, D. J. Lock and key colloids. *Nature* **464**, 575–578 (2010).

[4] Gerbode, S. J. *et al.* Glassy dislocation dynamics in 2D colloidal dimer crystals. *Phys. Rev. Lett.* **105**, 078301 (2010).

[5] Sanchez, T., Chen, D. T. N., DeCamp, S. J., Heymann, M. & Dogic, Z. Spontaneous motion in hierarchically assembled active matter. *Nature* **491**, 431–434 (2012).

[6] Glotzer, S. C. & Solomon, M. J. Anisotropy of building blocks and their assembly into complex structures. *Nat. Mater.* **6**, 557–562 (2007).

[7] Ashurst, W. T. & Hoover, W. G. Microscopic fracture studies in the two-dimensional triangular lattice. *Phys. Rev. B* **14**, 1465–1473 (1976).

[8] Succi, S. *The lattice Boltzmann equation for fluid dynamics and beyond* (Oxford University Press, New York, 2001).

[9] Alexeev, A., Verberg, R. & Balazs, A. C. Modeling the motion of microcapsules on compliant polymeric surfaces. *Macromolecules* **38**, 10244–10260 (2005).

[10] Smith, K. A., Alexeev, A., Verberg, R. & Balazs, A. C. Designing a simple ratcheting system to sort microcapsules by mechanical properties. *Langmuir* **22**, 6739–6742 (2006).

[11] Kolmakov, G. V., Yashin, V. V., Levitan, S. P. & Balazs, A. C. Designing communicating colonies of biomimetic microcapsules. *Proc. Nat. Acad. Sci. U.S.A.* **107**, 12417–12422 (2010).

[12] Dayal, P., Kuksenok, O., Bhattacharya, A. & Balazs, A. C. Chemically-mediated communication in self-oscillating, biomimetic cilia. *J. Mater. Chem.* **22**, 241–250 (2012).

[13] Wu, T.-H., Guo, R.-S., He, G.-W., Liu, Y.-M. & Qi, D. Simulation of swimming of a flexible filament using the generalized lattice-spring lattice-Boltzmann method. *J. Theor. Biol.* **349**, 1–11 (2014).

[14] Ladd, A. J. C. & Verberg, R. Lattice-Boltzmann simulations of particle-fluid suspensions. *J. Stat. Phys.* **104**, 1191–1251 (2001).

[15] Ladd, A. J. C., Kinney, J. H. & Breunig, T. M. Deformation and failure in cellular materials. *Phys. Rev.*

E **55**, 3271–3275 (1997).

[16] French, R. H. *et al.* Long range interactions in nanoscale science. *Rev. Mod. Phys.* **82**, 1887–1944 (2010).

[17] Villani, D. D., Yung, K. W. & Landecker, P. B. An analytic solution for the force between two magnetic dipoles. *Magnetic and Electrical Separation* **9**, 39–52 (1998).

[18] Landau, L. D. & Lifshitz, E. M. *Electrodynamics of continuous media*, vol. 8 of *Course of Theoretical Physics* (Butterworth and Heinemann, Oxford, 1987), 2nd edn.

[19] Dreyfus, R. *et al.* Microscopic artificial swimmers. *Nature* **437**, 862–865 (2005).

[20] Gaffney, E. A., Gadêlha, H., Smith, D. J., Blake, J. R. & Kirkman-Brown, J. C. Mammalian sperm motility: observation and theory. *Annu. Rev. Fluid Mech.* **43**, 501–528 (2011).

[21] Kokot, G., Piet, D., Whitesides, G. M., Aranson, I. S. & Snezhko, A. Emergence of reconfigurable wires and spinners via dynamic self-assembly. *Sci. Rep.* **5**, 9528 (2015).

[22] Lowe, C. P. Dynamics of filaments: modelling the dynamics of driven microfilaments. *Phil. Trans. R. Soc. Lond. B* **358**, 1543–1550 (2003).

[23] Budinski, K. G. & Budinski, M. K. *Engineering materials: properties and selection* (Prentice Hall, Columbus, 2004), 8th edn.

[24] Wiggins, C. H. & Goldstein, R. E. Flexive and propulsive dynamics of elastica at low Reynolds number. *Phys. Rev. Lett.* **80**, 3879–3882 (1998).

[25] Camalet, S., Jülicher, F. & Prost, J. Self-organized beating and swimming of internally driven filaments. *Phys. Rev. Lett.* **82**, 1590–1593 (1999).

[26] Purcell, E. M. Life at low Reynolds number. *Am. J. Phys.* **45**, 3–11 (1977).

[27] Wen, W., Kun, F., Pál, K. F., Zheng, D. W. & Tu, K. N. Aggregation kinetics and stability of structures formed by magnetic microspheres. *Phys. Rev. E* **59**, R4758–R4761 (1999).

[28] Tlusty, T. & Safran, S. A. Defect-induced phase separation in dipolar fluids. *Science* **290**, 1328–1331 (2000).

[29] Tripp, S. L., Pusztay, S. V., Ribbe, A. E. & Wei, A. Self-assembly of cobalt nanoparticle rings. *JACS* **124**, 7914–7915 (2002).

[30] Schmidle, H., Hall, C. K., Velev, O. D. & Klapp, S. H. L. Phase diagram of two-dimensional systems of dipole-like colloids. *Soft Matter* **8**, 1521–1531 (2012).
